# Exploring Dietary Factors and Esophageal Adenocarcinoma: Insights From Mendelian Randomization Study

**DOI:** 10.1002/fsn3.4527

**Published:** 2024-10-18

**Authors:** Xin Liu, Wenwen Yang, Yanjiang Yang

**Affiliations:** ^1^ Department of Intensive Care Medicine The First Hospital of Lanzhou University Lanzhou China; ^2^ The First Clinical Medical College Lanzhou University Lanzhou Gansu Province China; ^3^ Department of Rheumatology and Immunology The people's Hospital of Qiandongnan Autonomous Prefecture Kaili Guizhou Province China; ^4^ Qiandongnan Hospital Affiliated Hospital of Guizhou Medical University Kaili Guizhou Province China

**Keywords:** dietary intake, esophageal adenocarcinoma, Mendelian randomization, the UK Biobank

## Abstract

Esophageal adenocarcinoma and diet are not well understood to be associated. We conducted Mendelian randomization analysis using 18 dietary factors as exposures (primarily including fruit consumption, vegetable consumption, alcohol consumption, meat consumption, tea intake, fish intake, etc.), with esophageal adenocarcinoma as the outcome. The IVW method was the leading method used for detecting causal links. Cochran's *Q* test was utilized to assess heterogeneity, the intercept of the MR‐Egger method was used to assess the presence of horizontal pleiotropy, and the existence of outliers was identified via the MR‐Presso method. This study identified that both alcohol intake frequency (OR = 1.375, *p* = 0.0216) and coffee intake (OR = 2.680, *p* = 0.0304) were linked to a heightened risk of esophageal adenocarcinoma, while raw vegetable/salad consumption (OR = 0.117, *p* = 0.0258) and dried fruit intake (OR = 0.229, *p* = 0.00235) were associated with a decreased risk of esophageal adenocarcinoma. After FDR correction, only dried fruit intake (*q* = 0.0423) remained statistically significant. However, there was no evidence linking the other 14 dietary variables to esophageal adenocarcinoma. This study observed that alcohol consumption and coffee intake increase the risk of esophageal adenocarcinoma, while the intake of dried fruits rather than fresh fruits and raw vegetable intake rather than cooked vegetable intake reduce the risk of esophageal adenocarcinoma. Other dietary factors were not associated with the risk of esophageal adenocarcinoma.

## Graphical abstract

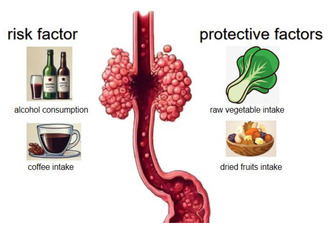

## 1 Introduction

More than 600,000 cases of esophageal cancer are identified worldwide each year, making it a serious global health concern (Sung et al. 2021). It is the sixth leading type of cancer globally and ranks as the eighth leading cause of cancer‐related fatalities (Sadat Yousefi et al. 2018). In Western countries, the most common histologic type of esophageal cancer is esophageal adenocarcinoma (Coleman, Xie, and Lagergren 2018). Some studies suggested that certain dietary factors may increase the risk of developing esophageal cancer. For example, some studies indicate that alcohol consumption, red meat consumption, and low intake of fruits and vegetables are major dietary risk factors for esophageal cancer (Ghosh and Jones 2022). However, some studies have yielded conflicting results (Hardikar et al. 2013; Jeurnink et al. 2012). A study discovered no direct relationship between alcohol intake and the risk of esophageal adenocarcinoma (Hardikar et al. 2013). A European prospective investigation found that the consumption of vegetables and fruits reduces the risk of esophageal squamous cell carcinoma, but has no effect on esophageal adenocarcinoma (Jeurnink et al. 2012). Therefore, the relationship between diet and esophageal adenocarcinoma remains incompletely understood. This study used Mendelian randomization (MR) analysis to investigate the impact of 18 dietary factors on esophageal adenocarcinoma, which holds significant implications for gaining deeper insights into the epidemiology of esophageal adenocarcinoma. In epidemiology and genetics, MR is a technique that uses genetic variants as instrumental variables (IVs) for evaluating causal links between exposures and outcomes. This method can help investigators in overcoming common observational research issues such as confounding and reverse causality (Sanderson et al. 2022).

## 2 Methods

MR is founded on the following assumptions: 1. IVs exhibit no direct association with the outcome; 2. IVs are strongly linked with the exposures; 3. IVs show no correlation with any potential confounding factor(s).

### 2.1 Data Origins

The IEU OpenG was project gathered and parsed data from the UK Biobank and previously released papers (The OpenGWAS project 2024). The GWAS data for esophageal adenocarcinoma as the outcome is sourced from the GWAS catalog (Sollis et al. 2023). Our study included 18 dietary factors as exposures, encompassing alcohol intake, meat consumption, fruit and vegetable intake, staple food consumption, and coffee and tea consumption. Further information regarding exposures is provided in Table 1 and Table S1. The information on how each dietary factor was measured is provided in Table S1. The data for esophageal adenocarcinoma as the outcome is sourced from the study conducted by Gharahkhani (2016), which shared its data on the GWAS catalog. The data used in this research was open, de‐identified, and anonymous. Thus, the Ethical Review Authority was not required to approve this study.

**TABLE 1:** Information on exposure and outcome datasets.

IEU GWAS id	Exposure or outcome	Identified SNPs	Participants included in analysis	*F*‐statistic
ieu‐b‐73	Alcoholic drinks per week	28	335,394 European‐descent individuals	99.170
ukb‐b‐5779	Alcohol intake frequency	84	462,346 European‐descent individuals	115.364
ukb‐b‐6324	Processed meat intake	21	461,981 European‐descent individuals	40.006
ukb‐b‐8006	Poultry intake	6	461,900 European‐descent individuals	24.445
ukb‐b‐2862	Beef intake	11	461,053 European‐descent individuals	26.941
ukb‐b‐17,627	Non‐oily fish intake	9	460,880 European‐descent individuals	26.378
ukb‐b‐2209	Oily fish intake	46	460,443 European‐descent individuals	38.093
ukb‐b‐5640	Pork intake	11	460,162 European‐descent individuals	19.162
ukb‐b‐14,179	Lamb/mutton intake	26	460,006 European‐descent individuals	20.355
ukb‐b‐11,348	Bread intake	25	452,236 European‐descent individuals	38.505
ukb‐b‐1489	Cheese intake	52	451,486 European‐descent individuals	44.973
ukb‐b‐8089	Cooked vegetable intake	13	448,651 European‐descent individuals	21.275
ukb‐b‐6066	Tea intake	33	447,485 European‐descent individuals	67.524
ukb‐b‐3881	Fresh fruit intake	46	446,462 European‐descent individuals	15.761
ukb‐b‐15,926	Cereal intake	34	441,640 European‐descent individuals	32.212
ukb‐b‐1996	Salad/raw vegetable intake	11	435,435 European‐descent individuals	16.939
ukb‐b‐5237	Coffee intake	34	428,860 European‐descent individuals	44.095
ukb‐b‐16,576	Dried fruit intake	35	421,764 European‐descent individuals	23.824
NA	esophageal adenocarcinoma (PMID:27527254)	N/A	4112 European‐descent cases and 17,159 European‐descent controls	N/A

*Note:* More information about exposures and outcome is available at the IEU OpenGWAS project (https://gwas.mrcieu.ac.uk/) and the GWAS catalog (https://www.ebi.ac.uk/gwas/home).

Abbreviations: GWAS: genome‐wide association studies; IEU: integrative epidemiology unit; NA: not applicable; SNPs: single‐nucleotide polymorphisms.

### 2.2 IVs Selection

In Mendelian randomization analysis, IVs were used to investigate the causal relationship between exposure factors and outcomes by acting as mediators. IVs commonly consist of genetic variants, with single‐nucleotide polymorphisms (SNPs) being among the extremely widely utilized. We selected SNPs that met the following criteria: significance level below genome‐wide threshold (*p* < 5 × 10^−8^), clumping window larger than 10,000 kb, and linkage disequilibrium level below 0.001 (r^2^ < 0.001). We used the *F*‐statistic to assess the robustness of the association between exposure and IVs. A value > 10 for the *F*‐statistic indicated a strong association.

### 2.3 Statistical Analysis

In this study, we used the inverse‐variance‐weighted (IVW) method as the principal analysis method for inferring causal relationships, supplemented by the MR‐Egger method and the weighted median (WM) method. The IVW method, which requires the absence of horizontal pleiotropy or balanced horizontal pleiotropy, possesses the highest capability for detecting causal relationships (Hartwig, Davey Smith, and Bowden 2017). We applied False Discovery Rate (FDR) control to adjust the *p*‐values derived from the IVW method, aiming to control the false discovery rate in multiple hypothesis testing (Glickman, Rao, and Schultz 2014). We used the MR‐Egger method to detect horizontal pleiotropy, as it allows for the presence of non‐zero intercepts. If < 50% of the IVs are invalid, the WM method is still effective (Hartwig, Davey Smith, and Bowden 2017). Even if all SNPs are invalid, the MR‐Egger method remains effective (Hartwig, Davey Smith, and Bowden 2017). We used Cochran's *Q*‐test to evaluate heterogeneity, whereby a *p*‐value under 0.05 from Cochran's *Q*‐test suggested heterogeneity. It is important to underscore that the existence of heterogeneity does not automatically imply the ineffectiveness of the IVW method. To identify any outliers, we utilized the MR‐PRESSO method. Upon detection, outliers were promptly eliminated, and the MR analysis was subsequently rerun. We used the TwoSampleMR package (Hemani et al. 2018) (version: 0.5.6) within R (version: 4.2.1) to perform all statistical analyses.

## 3 Result

We used 18 dietary factors (each with a participant population exceeding 300,000) as exposures and analyzed their impact on esophageal adenocarcinoma (4112 cases and 17,159 controls). The SNP count in this study varied from 6 to 84, with the *F*‐statistics for each SNP exceeding 10. Specifically, the *F*‐statistics for the 18 exposures varied from 15.761 to 115.364. *F*‐statistic exceeding 10 indicates sufficient power for causal inference to support the presence of a causal relationship. Additional information on dietary factors, esophageal adenocarcinoma, and the *F*‐statistics is provided in Table 1.

In this analysis of the impact of non‐oily fish intake, poultry intake, cooked vegetable intake, oily fish intake, and beef intake on esophageal adenocarcinoma, we detected outliers. The results after excluding outliers (if any) are presented in Table 2. As the IVW method is the most sensitive method for detecting causal relationships, we primarily rely on the IVW method for causal inference. Therefore, Table 2 mainly presents the results of the IVW method. Results from the WM method, MR‐Egger method, outlier situation, and analyses before and after outlier exclusion are presented in Table S2. In all analyses, no horizontal pleiotropy was detected, indicating the effectiveness of the IVW method. This study found causal relationships between four dietary factors and esophageal adenocarcinoma in the IVW method. Specifically, this study identified that both alcohol intake frequency (OR = 1.375, *p* = 0.0216) and coffee intake (OR = 2.680, *p* = 0.0304) were linked to a heightened risk of esophageal adenocarcinoma, while raw vegetable/salad consumption (OR = 0.117, *p* = 0.0258) and dried fruit intake (OR = 0.229, *p* = 0.00235) were associated with a decreased risk of esophageal adenocarcinoma. After FDR correction, only dried fruit intake (*q* = 0.0423) remained statistically significant. It must be noted that this study revealed that alcohol intake frequency increases the risk of esophageal adenocarcinoma, while weekly alcohol intake (OR = 1.715, *p* = 0.117) does not. Our study also observed that the intake of raw vegetable and dried fruit reduce the risk of esophageal adenocarcinoma, whereas cooked vegetable intake (OR = 1.223, *p* = 0.827) and fresh fruit intake (OR = 1.341, *p* = 0.600) do not. Additional information is provided in Table 2 and Table S2.

**TABLE 2:** The results of the IVW method on the influence of dietary factors on esophageal adenocarcinoma.

	Phenocode in IEU OpenGWAS project	Exposure	Used SNPs	Inverse variance weighted method			Cochrane's *Q* test		Pleiotropy		
				OR (95% CI)	*p*	*q*	*Q*	*p*	MR‐Egger intercept	SE	*p*
1	ieu‐b‐73	Alcoholic drinks per week	28	1.715 (0.874–3.363)	0.117	0.363	40.228	0.0488	0.0152	0.0112	0.186
2	ukb‐b‐5779	Alcohol intake frequency	84	1.375 (1.048–1.806)	0.0216	0.137	88.791	0.312	0.0124	0.00745	0.0988
3	ukb‐b‐6324	Processed meat intake	21	0.814 (0.329–2.018)	0.657	0.925	18.663	0.544	0.0335	0.0334	0.328
4	ukb‐b‐8006	Poultry intake	6	10.607 (0.536–210.044)	0.121	0.363	9.681	0.0848	−0.321	0.475	0.537
5	ukb‐b‐2862	Beef intake	11	1.000 (0.115–8.672)	1	1	19.266	0.037	−0.0663	0.074	0.394
6	ukb‐b‐17,627	Non‐oily fish intake	9	1.614 (0.190–13.741)	0.661	0.925	12.367	0.136	0.0356	0.0604	0.574
7	ukb‐b‐2209	Oily fish intake	47	0.975 (0.492–1.931)	0.942	0.997	54.133	0.165	−0.0295	0.0203	0.152
8	ukb‐b‐5640	Pork intake	11	0.619 (0.104–3.696)	0.599	0.925	5.456	0.859	−0.0314	0.0615	0.622
9	ukb‐b‐14,179	Lamb/mutton intake	26	0.834 (0.245–2.838)	0.771	0.925	28.776	0.273	0.0105	0.0296	0.725
10	ukb‐b‐11,348	Bread intake	25	1.205 (0.384–3.780)	0.749	0.925	45.333	0.00532	0.00824	0.0396	0.837
11	ukb‐b‐1489	Cheese intake	52	0.785 (0.405–1.520)	0.472	0.925	78.784	0.00753	−0.0141	0.0235	0.551
12	ukb‐b‐8089	Cooked vegetable intake	13	1.223 (0.199–7.506)	0.827	0.93	16.301	0.178	0.196	0.0892	0.0506
13	ukb‐b‐6066	Tea intake	33	1.125 (0.572–2.214)	0.732	0.925	46.147	0.0505	−0.00517	0.015	0.733
14	ukb‐b‐3881	Fresh fruit intake	46	1.341 (0.447–4.032)	0.6	0.925	59.429	0.0732	−0.00232	0.0183	0.899
15	ukb‐b‐15,926	Cereal intake	34	0.548 (0.212–1.418)	0.215	0.553	49.118	0.0352	0.021	0.0289	0.472
16	ukb‐b‐1996	Salad/raw vegetable intake	11	0.117 (0.0178–0.773)	0.0258	0.137	9.35	0.499	−0.0902	0.0516	0.114
17	ukb‐b‐5237	Coffee intake	34	2.680 (1.098–6.543)	0.0304	0.137	57.887	0.00471	0.00145	0.0152	0.924
18	ukb‐b‐16,576	Dried fruit intake	35	0.229 (0.0887–0.592)	0.00235	0.0423	41.002	0.19	−0.0164	0.0276	0.557

*Note:* The impact of dietary factors on esophageal adenocarcinoma. The IVW method, known for its heightened sensitivity in detecting causality, serves as our primary tool to ascertain the presence of a causal relationship. The MR‐Egger method is also used for detecting horizontal pleiotropy due to its allowance for the presence of non‐zero intercepts (The columns in the table corresponding to “MR‐Egger intercept”, “SE”, and “*p*‐value”), a pval below 0.05 suggests the existence of horizontal pleiotropy. *q* value: The *p* value post FDR method (Benjamini and Hochberg) corrected.

Abbreviations: CI: confidence interval; OR: odds ratio; SNPs: single‐nucleotide polymorphisms.

Additionally, our study observed that processed meat intake (OR = 0.814, *p* = 0.657), beef intake (OR = 1.000, *p* = 1.000), poultry intake (OR = 10.607, *p* = 0.121), oily fish consumption (OR = 0.975, *p* = 0.942), pork intake (OR = 0.619, *p* = 0.599), non‐oily fish intake (OR = 1.614, *p* = 0.661), mutton/lamb intake (OR = 0.834, *p* = 0.771), cheese intake (OR = 0.785, *p* = 0.472), bread intake (OR = 1.205, *p* = 0.749), tea intake (OR = 1.125, *p* = 0.732), and cereal intake (OR = 0.548, *p* = 0.215) were not associated with esophageal adenocarcinoma. Additional details are provided in Table 2 and Table S2.

## 4 Discussion

After analyzing the effects of 18 dietary factors on esophageal adenocarcinoma, this study observed that alcohol intake frequency and coffee consumption were risk factors for esophageal adenocarcinoma, while the intake of raw vegetable and dried fruit were protective factors. In addition to these findings, we believe the following observations should also be noted:

First, we observed that alcohol intake frequency (OR = 1.375, *p* = 0.0216) is associated with an increased risk of esophageal adenocarcinoma, whereas weekly alcohol intake (OR = 1.715, *p* = 0.117) is not linked to the risk of esophageal adenocarcinoma. The relationship between alcohol intake and esophageal adenocarcinoma has been examined in certain studies (Hardikar et al. 2013; Coleman, Xie, and Lagergren 2018; Chen et al. 2011). Some of these studies observed no direct correlation between alcohol intake and the risk of esophageal adenocarcinoma (Hardikar et al. 2013; Coleman, Xie, and Lagergren 2018). However, a study from Japan found that alcohol consumption increases the risk of developing esophageal cancer (Oze et al. 2011). A study in North China identified that drinking alcohol increases the risk of esophageal adenocarcinoma (Chen et al. 2011). Alcohol intake is a firmly established risk element for esophageal squamous cell carcinoma (Zambon et al. 2000; Freedman et al. 2007). Our study found that alcohol intake frequency is a risk factor for esophageal adenocarcinoma, while weekly alcohol intake is not. We speculate that this difference may be due to the fact that alcohol indeed increases the risk of esophageal adenocarcinoma, but the effect of alcohol intake frequency outweighs that of weekly alcohol intake (as we found that both the OR values for alcohol intake frequency and weekly alcohol intake in relation to esophageal adenocarcinoma were > 1).

Second, we found that intake of dried fruit (OR = 0.229, *p* = 0.00235) and raw vegetables (OR = 0.117, *p* = 0.0258) decreases the risk of esophageal adenocarcinoma, whereas consumption of fresh fruit (OR = 1.341, *p* = 0.600) and cooked vegetables (OR = 1.223, *p* = 0.827) does not demonstrate such an effect. A European prospective investigation found that the intake of vegetables and fruits reduces the risk of esophageal squamous cell carcinoma, but has no effect on esophageal adenocarcinoma (Jeurnink et al. 2012). There is evidence to suggest that increasing the consumption of dried fruit may have a potential impact on cancer risk (Mossine, Mawhinney, and Giovannucci 2020). A cohort study from the Netherlands (Botterweck, van den Brandt, and Goldbohm 1998) and two case–control studies from Spain (González et al. 1991) and Turkey (Yassibaş, Arslan, and Yalçin 2012) respectively found that the intake of dried fruit reduces the risk of stomach cancer, while fresh fruit intake is not associated with stomach cancer risk. Comparative analysis suggests that adenocarcinoma of the esophagogastric junction and stomach cancer are more likely to be classified as a group of tumors because of their many similarities (Suh et al. 2012). Therefore, esophageal adenocarcinoma and stomach cancer may also share similar risk factors and protective factors, enhancing the reliability of our study results given their significant resemblance to previous research findings. Currently, several studies have investigated the relationship between vegetable consumption and esophageal adenocarcinoma (Jeurnink et al. 2012; Li et al. 2014). The intake of vegetables and the incidence of esophageal adenocarcinoma are significantly inversely correlated, based on a meta‐analysis of observational studies (Li et al. 2014). Another study found that vegetable intake is not associated with esophageal adenocarcinoma or gastric cancer (Jeurnink et al. 2012). It is noteworthy that their study did not differentiate whether vegetables were cooked or not, which could lead to different effects. Our study found that vegetable consumption can lower the risk of esophageal adenocarcinoma, but whether vegetables were cooked may have yielded entirely different effects. The cooking process can influence the overall antioxidant capacity and chemical composition of vegetables, which may affect their nutritional value (Pellegrini et al. 2010). This variation may be the reason for the different effects on esophageal adenocarcinoma between the consumption of raw vegetables and cooked vegetables. A meta‐analysis found no association between coffee consumption and esophageal cancer in Western populations (Zhang, Zhou, and Hao 2018). However, this study found that coffee consumption increases the risk of esophageal adenocarcinoma. We believe that due to the limited research on the relationship between coffee intake and esophageal adenocarcinoma, more studies are required to elucidate their relationship.

The mechanism currently linking dietary factors to esophageal adenocarcinoma remains incompletely understood. We hypothesize that dietary factors may influence esophageal adenocarcinoma through two distinct pathways. First, the esophagus serves as a direct conduit for food, allowing ingested substances to pass through or briefly reside within it. This enables direct exposure of the esophagus to the composition of food items (such as nutrients, nitrosamines, fats, proteins, and vitamins) or to substances induced by high‐temperature cooking, baking, and frying processes. This direct exposure may constitute an important pathway linking dietary factors to esophageal adenocarcinoma. Second, another possible pathway is that substances in the diet are absorbed and then act on the esophagus through blood circulation, affecting the incidence of esophageal adenocarcinoma.

This MR analysis should be regarded more cautiously, given that it revealed associations between alcohol consumption frequency, raw vegetable consumption, coffee consumption, and dry fruit consumption, and the incidence of esophageal adenocarcinoma. First, the findings from the MR analysis indicated that prolonged exposure to certain dietary factors may lead to esophageal adenocarcinoma, thus short‐term impacts may lack clinical relevance. Second, inability of MR to discern causal relationships across varying timeframes presents another noteworthy limitation. For instance, a magnetic resonance study identified a correlation between multiple sclerosis and vitamin D (Mokry et al. 2015); however, this association was only evident during childhood or earlier (Chaudhuri 2005). Nevertheless, our study analyzed the relationship between 18 dietary factors and esophageal adenocarcinoma, which is of paramount importance for exploring the epidemiology of esophageal adenocarcinoma.

Certainly, inherent limitations are inevitable within this study. First, we are unable to differentiate between the effects of various food combinations or to further split distinct types of dietary intake. For example, we found that the intake of dried fruits reduces the risk of esophageal adenocarcinoma, but we are unable to identify which specific type or types of dried fruits have this effect. Second, due to the absence of summary‐level GWAS data stratified by gender and age, we are unable to perform gender‐stratified and age‐stratified analyses. Third, it is unclear whether the results can be applied to other populations because the exposure and outcome data were obtained exclusively from European populations.

## 5 Conclusion

This study observed that alcohol consumption and coffee intake increase the risk of esophageal adenocarcinoma, while the intake of dried fruits rather than fresh fruits and raw vegetable intake rather than cooked vegetable intake reduces the risk of esophageal adenocarcinoma. Other dietary factors were not associated with the risk of esophageal adenocarcinoma.

## Author Contributions

**Xin Liu:** conceptualization (equal), investigation (equal), project administration (equal), resources (equal), writing – review and editing (equal). **Wenwen Yang:** conceptualization (equal), methodology (equal), project administration (equal), validation (equal). **Yanjiang Yang:** conceptualization (equal), data curation (equal), formal analysis (equal), funding acquisition (equal), investigation (equal), methodology (equal), project administration (equal), resources (equal), software (equal), supervision (equal), validation (equal), visualization (equal), writing – original draft (equal), writing – review and editing (equal).

## Conflicts of Interest

The authors declare no conflicts of interest.

## Supporting information


Tables S1‐S2.

## Data Availability

All GWAS data used in this study are available in the IEU open GWAS project (https://gwas.mrcieu.ac.uk/) and the GWAS catalog (https://ftp.ebi.ac.uk/pub/databases/gwas/summary_statistics/GCST003001‐GCST004000/GCST003739/).
